# Predicting potential distribution of *Ziziphus spinosa* (Bunge) H.H. Hu ex F.H. Chen in China under climate change scenarios

**DOI:** 10.1002/ece3.8629

**Published:** 2022-02-17

**Authors:** Qian Zhao, Ze‐Yuan Mi, Chan Lu, Xin‐Fei Zhang, Li‐Jun Chen, Shi‐Qiang Wang, Jun‐Feng Niu, Zhe‐Zhi Wang

**Affiliations:** ^1^ National Engineering Laboratory for Resource Development of Endangered Crude Drugs in Northwest China Shaanxi Normal University Xi’an China; ^2^ Key Laboratory of Medicinal Resources and Natural Pharmaceutical Chemistry (Shaanxi Normal University) The Ministry of Education Xi’an China; ^3^ College of Life Sciences Shaanxi Normal University Xi’an China

**Keywords:** ecological niche modeling, maximum entropy, prediction of suitable region, *Ziziphus spinosa* (Bunge) H.H. Hu ex F.H. Chen

## Abstract

*Ziziphus spinosa* (Bunge) H.H. Hu ex F.H. Chen is a woody plant species of the family Rhamnaceae (order Rhamnales) that possesses high nutritional and medicinal value. Predicting the effects of climate change on the distribution of *Z*. *spinosa* is of great significance for the investigation, protection, and exploitation of this germplasm resource. For this study, optimized maximum entropy models were employed to predict the distribution patterns and changes of its present (1970–2000) and future (2050s, 2070s, and 2090s) potential suitable regions in China under multiple climate scenarios (SSP1‐2.6, SSP2‐4.5, SSP3‐7.0 & SSP5‐8.5). The results revealed that the total area of the present potential suitable region for *Z*. *spinosa* is 162.60 × 10^4^ km^2^, which accounts for 16.94% of China's territory. Within this area, the regions having low, medium, and high suitability were 80.14 × 10^4^ km^2^, 81.50 × 10^4^ km^2^, and 0.96 × 10^4^ km^2^, respectively, with the high suitability regions being distributed primarily in Shanxi, Hebei, and Beijing Provinces. Except for SSP‐1‐2.6‐2070s, SSP‐5‐8.5‐2070s, and SSP‐5‐8.5‐2090s, the suitable areas for *Z*. *spinosa* in the future increased to different degrees. Meanwhile, considering the distribution of *Z*. *spinosa* during different periods and under different climate scenarios, our study predicted that the low impact areas of *Z*. *spinosa* were mainly restricted to Shanxi, Shaanxi, Ningxia, Gansu, Liaoning, Inner Mongolia, and Jilin Provinces. The results of core distributional shifts showed that, except for SSP1‐2.6, the center of the potential suitable region of *Z*. *spinosa* exhibited a trend of gradually shifting to the northwest.

## Graphical abstract

Predicting the effects of climate change on the distribution of *Ziziphus spinosa* is of great significance for the investigation, protection, and exploitation of this germplasm resource. For this study, optimized maximum entropy models were employed to predict the distribution patterns and changes of its present (1970–2000) and future (2050s, 2070s, and 2090s) potential suitable regions in China under multiple climate scenarios (SSP1‐2.6, SSP2‐4.5, SSP3‐7.0, and SSP5‐8.5).

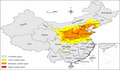

## 1 INTRODUCTION

Climate change is considered to be a key factor in altering the geographical distribution of species in the 21st century (Record et al., 2013; Santos‐Hernández et al., 2021; Waltari et al., 2007). According to the Fifth Assessment Report (AR5) of the Intergovernmental Panel on Climate Change (IPCC), the average global surface temperature is expected to rise by 0.3–4.8°C by the end of the 21st century due to the continuous increases in greenhouse gas emissions (Braunisch et al., 2013; Cahill et al., 2013; Carroll et al., 2010). In response to this warming trend, many studies have suggested that species would change their currently suitable habitats in response to changes in environmental conditions, particularly as species distribution increases in elevation and migrates to northern latitudes (Heikkinen et al., 2006; Kujala et al., 2013; Qiu et al., 2011; Schweiger et al., 2008). In addition to changes in spatial habitats, climate change is modifying sensitive ecological responses, including flowering periods and the duration of growing seasons (Hampe et al., 2013; Wang et al., 2013).

With the emergence of novel computational statistics technologies and the development of the Global Information System (GIS), direct correlations between environmental factors (e.g., climate, topography, meteorological data, species data) have become possible, which is extensively used in ecological applications (Ye et al., 2020). Ecological niche models (ENMs), also known as species distribution models (SDMs) (Brown, 2014; Brown et al., 2017), are employed to estimate the relationships between species presence and environmental factors through the extrapolation of multiple algorithms at multiple temporal and spatial scales (Conolly et al., 2012), which can be used to predict the potential distribution of species (Conolly et al., 2012). Over the last few decades, ENMs have played an important role in predicting the potential geographic distribution of species in the context of climate change, and have been broadly used in the domains of biology, ecology, biogeography, evolutionary biology, and species conservation (Araújo & Guisan, 2006). Among various ENM/SDM methodologies, maximum entropy (MAXENT) modeling has exhibited a better predictive ability, to become one of the most widely used models at present (Phillips et al., 2017; Radosavljevic & Anderson, 2014; Zeng et al., 2016). To date, the MAXENT model has been used to predict trends in the potential habitats of many plant species (particularly endangered species), such as *Mimusops laurifolia* (Forssk.) Friis (Hall et al., 2010) and *Semiliquidambar cathayensis* H.T. Chang (Ye et al., 2020).

*Ziziphus spinosa* (Bunge) H.H. Hu ex F.H. Chen is a woody plant within the family Rhamnales (order Rhamnaceae). It is native to Liaoning, Hebei, Shandong, Shanxi, Shaanxi, Henan, Inner Mongolia, Gansu, Xinjiang, Beijing, and other northern Provinces of China, which generally grows in mountainous, hilly, or plain areas with elevations of <1,700 m (Wang et al., 2021). *Ziziphus spinosa* possesses high nutritional, economic, and medicinal value, as its pulp is rich in sugars, acids, proteins, and vitamins, has a long flowering period, and can be a source of nectar. Moreover, Ziziphi Spinosae Semen (i.e., dry mature seeds of *Z*. *spinosa*) has such functions as nourishing the heart and liver, and treating insomnia (Li et al., 2021; Song et al., 2020). The past decade has witnessed an increasingly high market demand and price for *Z*. *spinosa* due to limited yields and supplies; thus, it has become urgent to promote its artificial planting and development.

To date, previous investigations of *Z*. *spinosa* have focused primarily on cultivation technologies and the pharmacological effects of Ziziphi Spinosae Semen (Li et al., 2021; Song et al., 2020). The present study employed an optimized maximum entropy model to predict and analyze the distribution areas of *Z. spinosa* under both present (1970‐2000) and future (2050s, 2070s and 2090s) climate scenarios (SSP1‐2.6, SSP2‐4.5, SSP3.70 & SSP5‐8.5).

## 2 MATERIALS AND METHODS

### 2.1 Collection and screening of sample data

**FIGURE 1:**
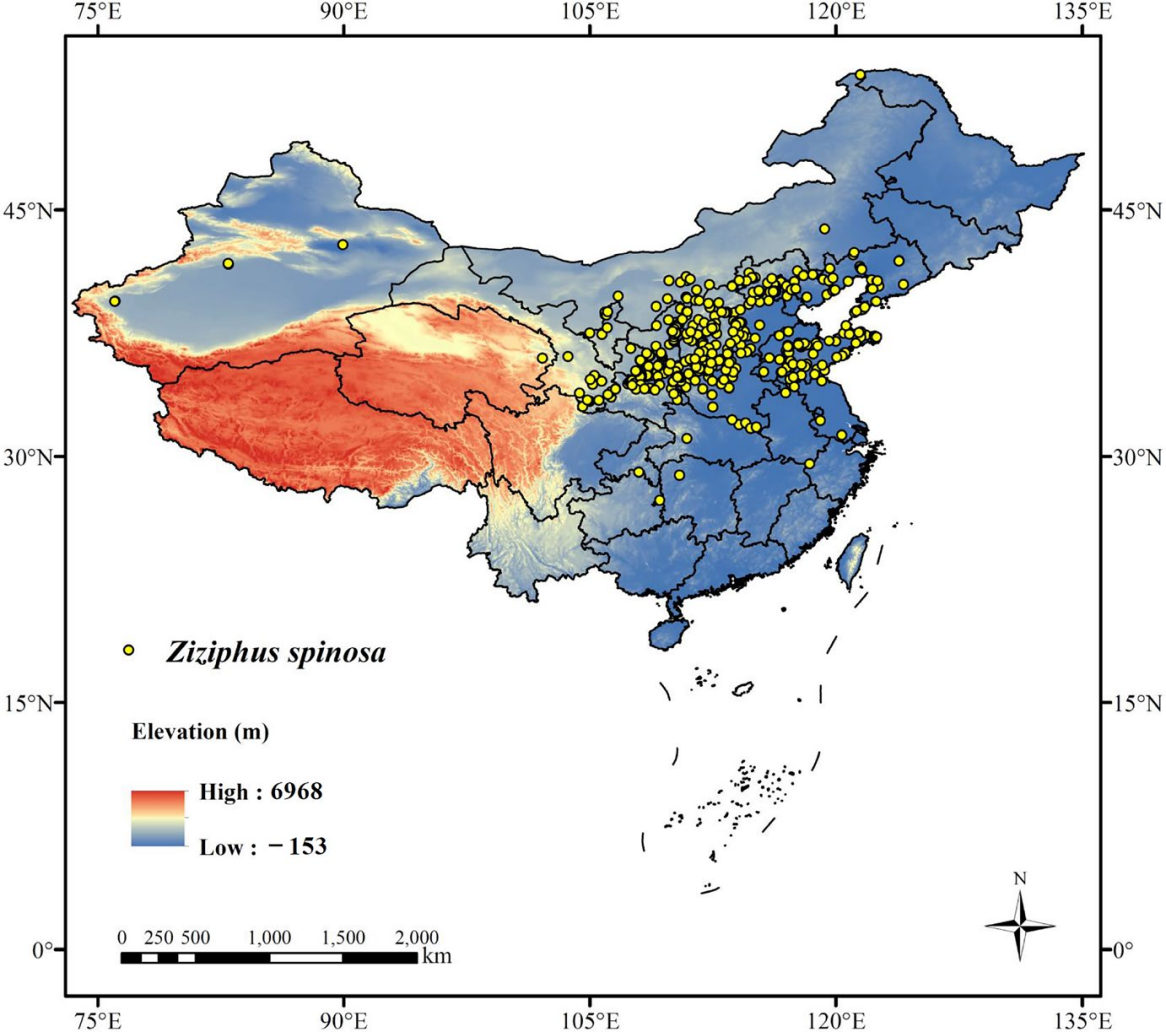
The occurrence data (406 points) of *Ziziphus spinosa* in China

Over the last 3 years (2019–2021), our research group conducted extensive field surveys in Shaanxi, Shanxi, Gansu, Hebei, Henan, and Shandong Provinces, and collected a total of 106 occurrence points. In addition, 321 occurrence points were obtained from the previously published literature and web databases [i.e., China National Knowledge Infrastructure/CNKI (https://www.cnki.net); Google Scholar (https://scholar.google.com); Chinese Virtual Herbarium/CVH (https://www.cvh.ac.cn); Chinese Field Herbarium/CFH (http://www.cfh.ac.cn), Plant Photo Bank of China/PPBC (http://www.ppbc.iplant.cn), and the Global Biodiversity Information Facility/GBIF (https://www.gbif.org)]. To minimize errors caused by clustering effects, each grid (2.5 arc‐minutes) retained only one occurrence point; thus, a final dataset of 406 occurrence points was employed for MAXENT modeling (Figure 1).

### 2.2 Variable environment screening and data processing

Bioclimatic variables are the major determinants of the ENMs of species and are frequently used for the development of plant ENMs (Tang et al., 2018). The set of 19 bioclimatic variables used in the present study were downloaded from the WorldClim website (https://www.worldclim.org), which involved a recent (1970–2000) and three future periods (2050s, 2070s, and 2090s). Considering the impacts of climate change scenarios on the accuracy of model development (Santos‐Hernández et al., 2021), we selected four shared socioeconomic pathways (SSPs; SSP1‐2.6, SSP2‐4.5, SSP3‐7.0, and SSP5‐8.5) for three general circulation models (GCMs; BCC‐CSM2‐MR, CNRM‐CM6‐1, and MIROC‐ES2L) in future climate data (Xu et al., 2020). Consequently, a total of 37 sets of bioclimatic data were included in this study, with one recent, and 36 future sets.

Firstly, the MAXENT pre‐model was developed based on species occurrence data and 19 bioclimatic factors, where the importance of each variable to the model was evaluated using the Jackknife method (Yang et al., 2013; Zeng et al., 2016; Zhang et al., 2020). Secondly, pairwise Pearson correlation coefficients (*R*) among the 19 bioclimatic factors were calculated using ENMTools v1.4.4 (Warren et al., 2010), and any pair of factors with |*R*| ≥ 0.8 were considered to be significantly correlated (Warren et al., 2010). Finally, for each pair of significantly correlated variables, only the ones with higher contributions to the model were retained (Cahill et al., 2013; Václavík & Meentemeyer, 2009, 2012; Warren et al., 2011; Wisz et al., 2013; Zeng et al., 2016).

### 2.3 Model establishment, optimization, and evaluation

Maxent v3.4.1 (Phillips et al., 2017) was used to construct the maximum entropy model for this study. Considering that the selection of general circulation models (GCMs) would lead to the uncertainty of the prediction results, we carried out arithmetic average processing on the prediction results of the three GCMs for the future periods. To ensure the probability of *Z*. *spinosa* distribution being close to the normal distribution, we selected 70% of the data for model training and the remaining data for model testing (Phillips & Dudík, 2008). The other major parameters were set as follows: <Maximum Iterations: 5000; Replicated Run Type: Crossvalidate; No. of Replicates: 10>.

In this study, the R package <kuenm> (Cobos et al., 2019) was employed to optimize the feature class (FC) and regularization multiplier (RM) of the MAXENT model. Firstly, the RM was set to 0.1–4 with each interval of 0.1, which resulted in a total of 40 RM values. Subsequently, the four FCs [Linear (L), Quadratic (Q), Hinge (H), Product (P)] in the MAXENT model were combined to form 15 FC combinations [i.e., L, P, Q, H, LP, LQ, LH, LPQ, LPH, LQH, LQH, LQH, LQH, PQ, PH, and QH]. Thus, a total of 600 parameter combinations were multiplied by the FC and RM. On the basis of optimal model determination, the model (OR_AICc) with a statistically significant omission rate that was lower than the threshold value (0.05), and a delta AICc value of less than 2 was selected (Liu et al., 2019; Ye et al., 2018).

### 2.4 Classification of suitable region and reliability test of model

The suitability of species habitats is typically represented by the value range 0–1, where the higher the value is, the more suitable a certain area is for the species to grow. The selection of thresholds has an important impact on the prediction of suitable regions of different grades, which affects the calculation of different suitable areas (Arenas‐Castro et al., 2020; Hu et al., 2020). Tang et al. (2018) proposed that a maximum test sensitivity plus specificity (MTSPS) threshold was superior to other threshold options in the grade division of suitable regions. Thus, MTSPS was employed as the threshold value for this study, and those areas with suitability values lower than MTSPS were considered unsuitable for the growth of the species. The suitability range between the MTSPS and 1 was subdivided into three equal parts, which corresponded to the low, moderate, and high suitability regions, respectively (Li et al., 2019; Wang et al., 2017). The area sizes of different suitable regions as well as their changes in different future periods were calculated by using DIVA‐GIS v7.5 (https://diva‐gis.org).

Following model construction, the area under the receiver operating characteristic curve (AUC) was used to evaluate the accuracy of the predictive model (Lobo et al., 2008). The mean AUC value was in the range of (0, 1), where AUC > 0.9 indicated that the model results were excellent and accurate (Warren & Seifert, 2010). Further, we considered the difference between the training AUC and the test AUC, where the smaller the absolute value of the difference, the higher the reliability of the model (Warren & Seifert, 2011).

### 2.5 Analysis of low impact areas

Low impact areas refer to those where species are relatively less affected by climate change, which can be projected by superposing the binary prediction maps of suitable regions in different periods and taking the completely overlapping parts (Pan et al., 2020). In DIVA‐GIS v7.5, the distribution maps of the potential suitable regions of different periods were overlaid to reclass the spatial units with suitability values greater than the MTSPS threshold as the suitable regions (Zhao, Zhang, et al., 2021). Those spatial units with suitability values lower than the MTSPS threshold were reclassed as unsuitable regions, which established the unsuitable and suitable matrices of *Z*. *spinosa*. Subsequently, the completely overlapping parts in the overlay layers were selected. After processing, the overlaid layers of different periods were imported into DIVA‐GIS v7.5, and the potential low impact areas of *Z*. *spinosa* were visualized. This study predicted the low impact areas of four different shared socioeconomic pathways (SSP1‐2.6, SSP2‐4.5, SSP3‐7.0 & SSP5‐8.5) in current and future periods (2050s, 2070s, and 2090s).

### 2.6 Analysis of spatial pattern changes

Spatial pattern changes refer to those of potential suitable regions of species across different periods, which could be obtained by superposing binary prediction maps of suitable regions during different periods (Santos‐Hernández et al., 2021; Wu et al., 2021; Zurell et al., 2020). For this study, we created a prediction chart of the spatial pattern changes of four different SSPs (SSP1‐2.6, SSP2‐4.5, SSP3‐7.0, and SSP5‐8.5) during current and future periods (2050s, 2070s, and 2090s), resulting in a total of 12 pattern change predictions. This was used to analyze the change rule of potential suitable regions of *Z*. *spinosa* in the recent period and three different future periods under various SSPs. In DIVA‐GIS v7.5, the distribution maps of potential suitable regions of different periods were superposed to establish both the unsuitability and suitability matrices of *Z*. *spinosa*. Based on the matrix table, the changes in spatial patterns of the suitable distribution regions under current and future climate change scenarios were further analyzed.

### 2.7 Core distributional shifts

SDMToolbox V2.4 toolkit (Brown, 2014; Brown et al., 2017) of ArcGIS v10.2 was employed to calculate the variation trend of different suitable regions for *Z*. *spinosa*, and the central points of different regions were compared. We considered the suitable region of *Z*. *spinosa* as a whole, simplified it to a vector particle, and used the change of the centroid position to reflect the size and direction of the suitable region of *Z*. *spinosa*. Finally, the SDMToolbox toolkit was used to track the centroid of *Z*. *spinosa*, to investigate the distribution of the centroid during different periods and under various climate conditions, and to evaluate the migration distance of suitable regions via longitude and latitude coordinates (Smith et al., 2019; Zurell et al., 2020).

## 3 RESULTS

### 3.1 Analysis of model accuracy and classification of suitable regions

Based on 406 distribution points and five bioclimatic variables, the MAXENT model was used to predict the distribution of potential suitable regions for *Z*. *spinosa*. The model optimization suggested that the optimal FC and RM were LQ and 0.1, respectively. The training AUC (AUC_TRAIN_) value of the model was 0.9526 ± 0.0019, and the test AUC (AUC_TEST_) value was 0.9500 ± 0.0052. The absolute value (|AUC_DIFF_|) of the difference between AUC_TRAIN_ and AUC_TEST_ was 0.0026. All of these results suggested an excellent model prediction accuracy.

Based on the MTSPS threshold (0.1613), the spatial units were subdivided as follows: 0–0.1613 Unsuitable region; 0.1614–0.4409 Low suitable region; 0.4410–0.7204 Moderate suitable region; 0.7204–1 High suitable region.

### 3.2 Contribution analysis of environmental variables

Following model optimization, five bioclimatic factors were used for the final model construction, i.e., temperature seasonality (BIO04), mean temperature of warmest quarter (BIO10), mean temperature of coldest quarter (BIO11), precipitation of warmest quarter (BIO18), and precipitation of coldest quarter (BIO19). Their percentage‐wise contributions to model construction were BIO11 (65.2%) > BIO18 (13.6%) > BIO19 (10.3%) > BIO10 (5.9%) > BIO04 (5.0%) (Table 1).

**TABLE 1:** Bioclimatic factors used for the final model development and their contributions

Code	Bioclimatic factors	Percent contribution (%)
BIO04	Temperature seasonality (standard deviation × 100)	5.0
BIO10	Mean temperature of warmest quarter (°C)	5.9
BIO11	Mean temperature of coldest quarter (°C)	65.2
BIO18	Precipitation of warmest quarter (mm)	13.6
BIO19	Precipitation of coldest quarter (mm)	10.3

### 3.3 Current potential suitable region

**FIGURE 2:**
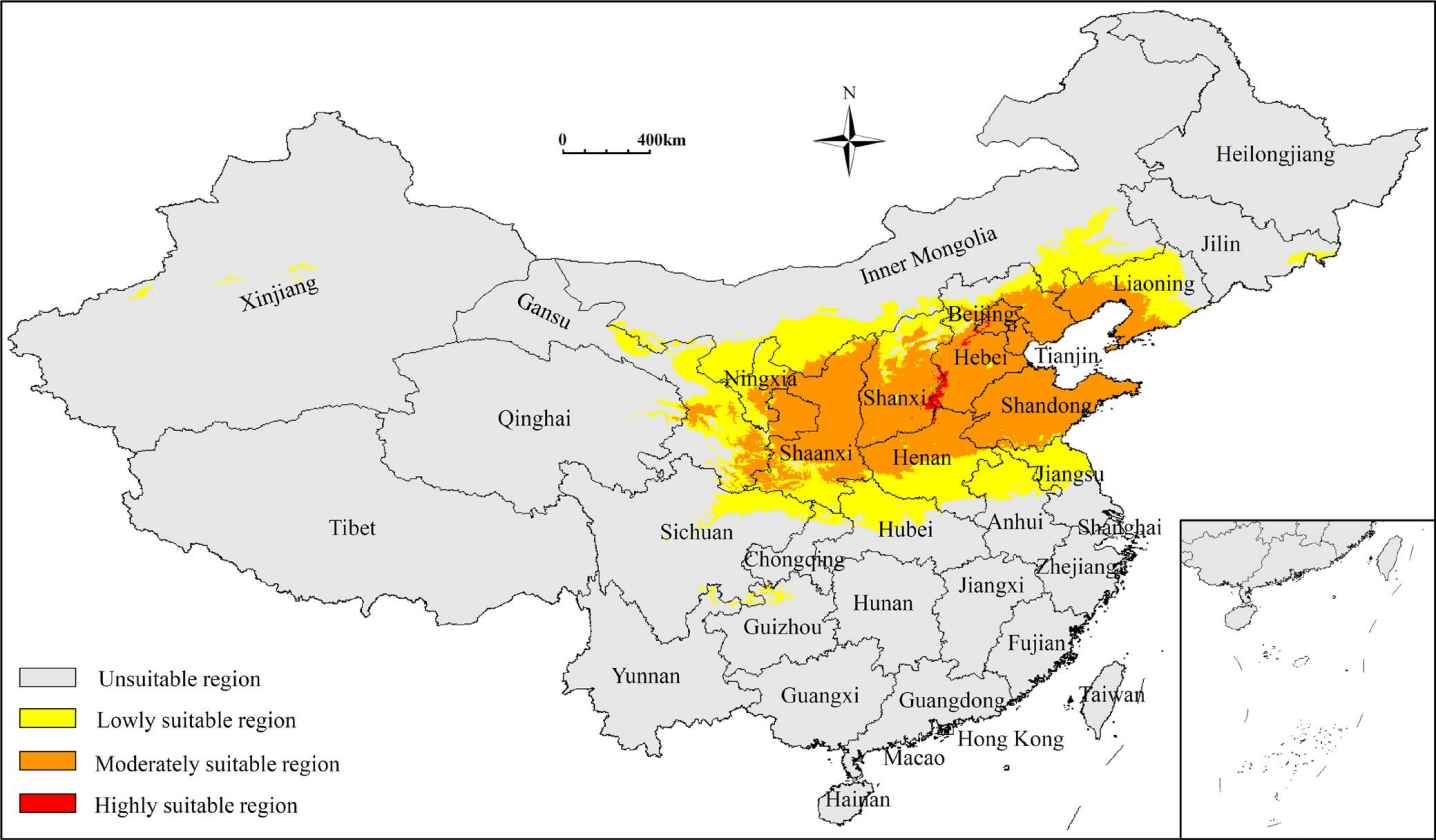
Predicted distribution of *Ziziphus spinosa* in China under current climate condition

The current potential suitable regions of *Z*. *spinosa* covered a total area of 162.60 × 10^4^ km^2^, and were mostly restricted to Beijing, Tianjin, Hebei, Shanxi, Inner Mongolia, Shaanxi, Ningxia, Henan, and Shandong Provinces. Additionally, fragmented distributions were also predicted in Gansu, Qinghai, Xinjiang, Hubei, Sichuan, Guizhou, Jilin, Jiangsu, and Anhui Provinces (Figure 2). The predicted areas of high, moderate, and low potential suitability regions were 0.96 × 10^4^ km^2^, 81.50 × 10^4^ km^2^, and 80.14 × 10^4^ km^2^, respectively. The highly suitable regions were mainly distributed in Shanxi (Changzhi and Jincheng), Hebei (Xingtai, Handan, and Baoding), and Beijing Provinces (Table 2).

**TABLE 2:** Predicted suitable areas under current and future climatic conditions

Decades		Predicted area (×10^4^ km^2^) and % of the corresponding current area							
		Total suitable region		Lowly suitable region		Moderately suitable region		Highly suitable region	
1970–2000		162.60	—	80.14	—	81.50	—	0.96	—
SSP1‐2.6	2050s	170.12	104.62%	102.49	127.88%	66.39	81.46%	1.24	128.88%
	2070s	160.76	98.86%	100.48	125.38%	58.96	72.34%	1.32	137.00%
	2090s	174.84	107.52%	106.14	132.44%	65.80	80.74%	2.89	301.26%
SSP2‐4.5	2050s	174.74	107.46%	106.74	133.19%	66.44	81.53%	1.55	161.55%
	2070s	174.56	107.36%	113.37	141.46%	60.25	73.93%	0.94	98.19%
	2090s	165.91	102.03%	107.57	134.22%	56.49	69.31%	1.85	192.78%
SSP3‐7.0	2050s	170.61	104.92%	107.44	134.06%	62.33	76.48%	0.83	86.82%
	2070s	179.51	110.40%	118.23	147.53%	59.64	73.18%	1.63	169.68%
	2090s	165.42	101.73%	115.10	143.62%	49.62	60.88%	0.70	72.92%
SSP5‐8.5	2050s	176.54	108.57%	111.25	138.82%	62.69	76.92%	2.60	270.76%
	2070s	149.88	92.17%	97.94	122.21%	51.09	62.68%	0.85	88.45%
	2090s	139.53	85.81%	108.25	135.07%	31.16	38.23%	0.12	12.82%

### 3.4 Future potential suitable regions

**FIGURE 3:**
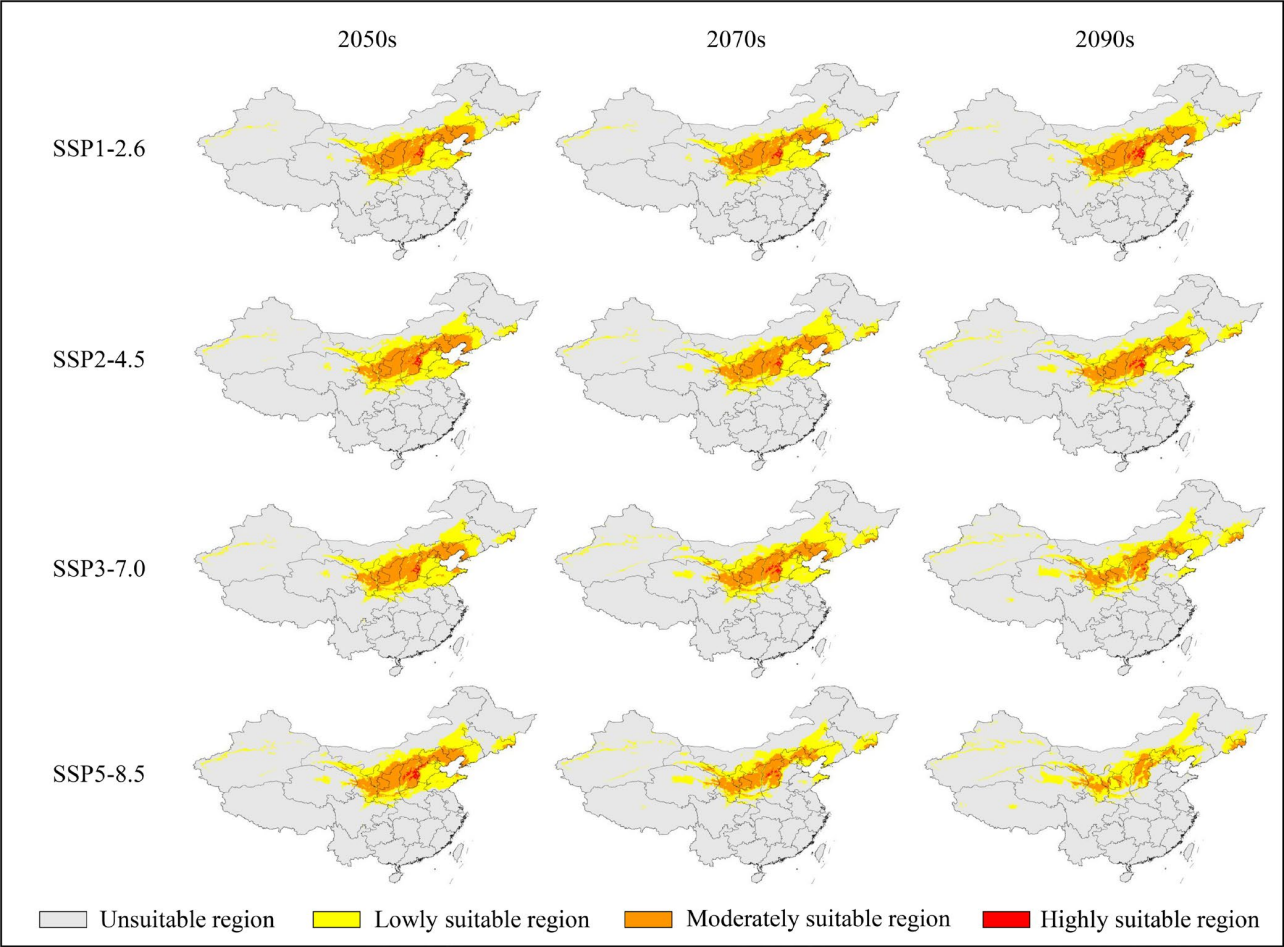
Predicted distribution of *Ziziphus spinosa* in China under future (2050s‐2090s) climatic scenarios

**FIGURE 4:**
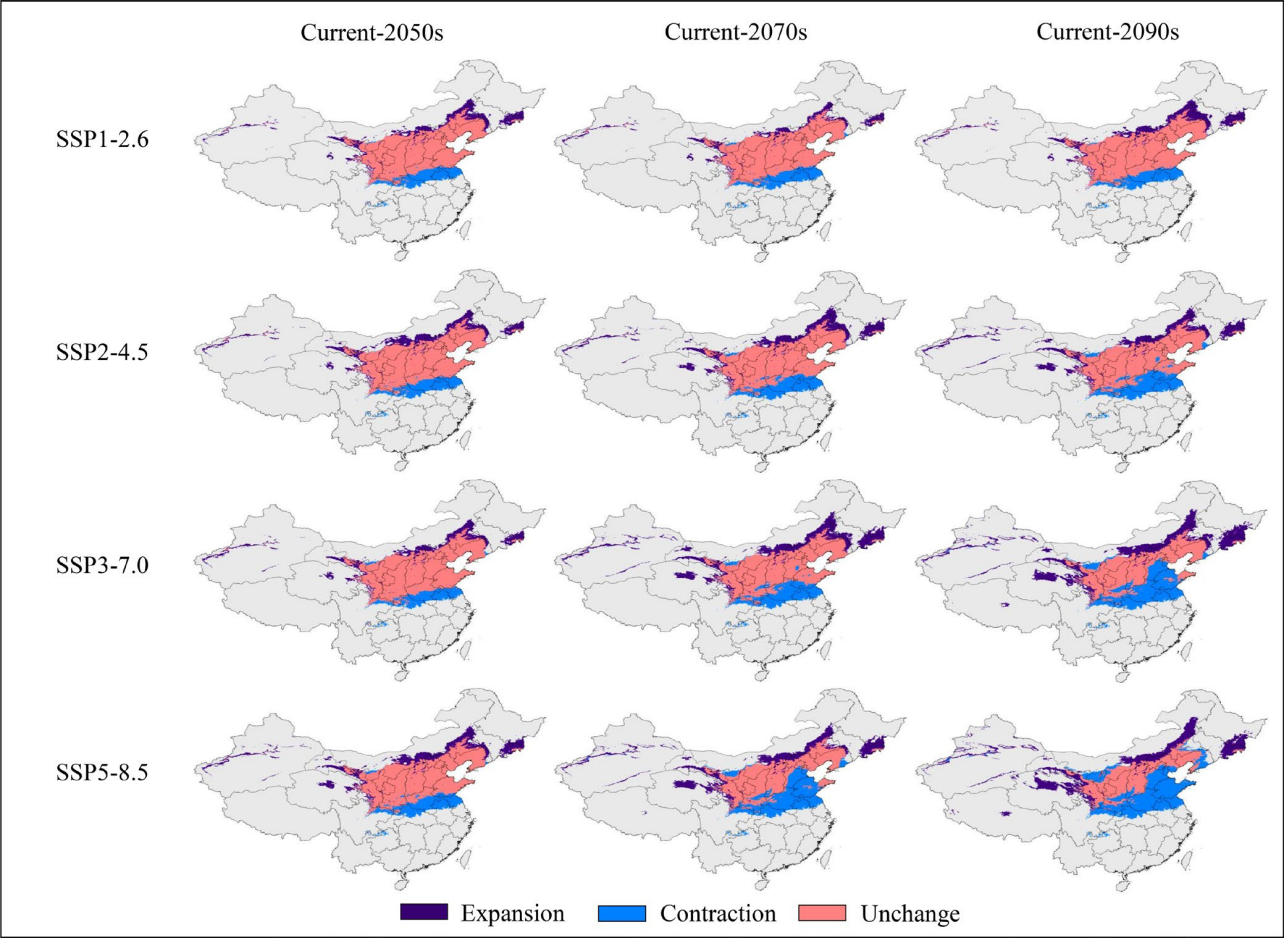
Changes of potential suitable areas of *Ziziphus spinosa* from current to future climatic conditions

**FIGURE 5:**
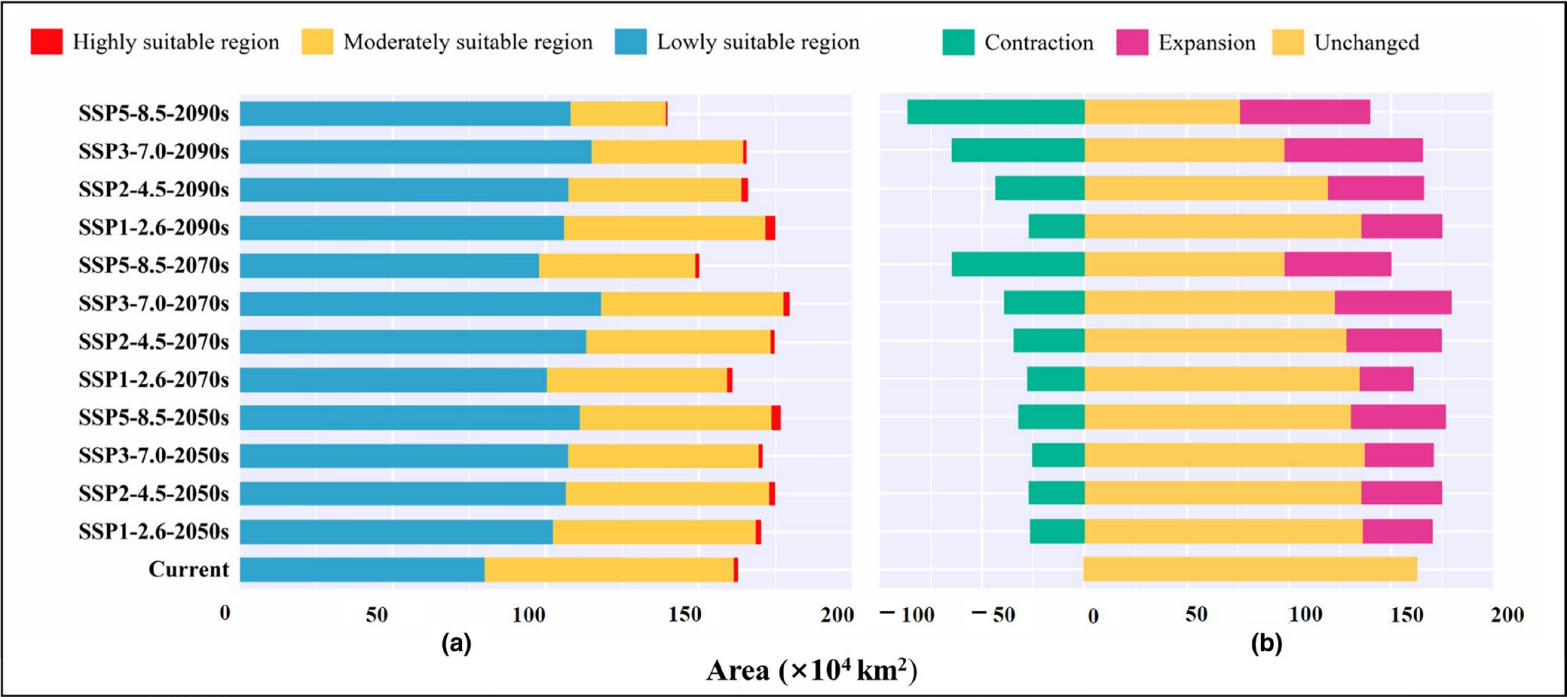
Areas (a) and changes (b) of habitats of different suitability for *Ziziphus spinosa* at different times in China

The distribution and changes in potential suitability regions for *Z*. *spinosa* during the three future periods differed under various climate scenarios; however, there were some similarities in the trends of changes (Figures 3, 4, 5; Tables 2 and 3). Under different climate scenarios, the area of low suitability regions increased significantly compared with its current value, while that of the moderate suitability regions decreased significantly. Conversely, the changing trends of total and highly suitable regions were not completely consistent under different climate scenarios (Tables 2 and 3).

**TABLE 3:** Change rates of *Ziziphus spinosa* suitable regions in different periods

Scenarios		Unchanged rate (%)	Expansion rate (%)	Contraction rate (%)
Current→2050s	SSP1‐2.6	83.87	20.76	16.13
	SSP2‐4.5	83.49	23.97	16.51
	SSP3‐7.0	84.56	20.36	15.44
	SSP5‐8.5	80.33	28.24	19.67
Current→2070s	SSP1‐2.6	82.98	15.89	17.02
	SSP2‐4.5	78.97	28.39	21.03
	SSP3‐7.0	75.47	34.93	23.92
	SSP5‐8.5	60.33	31.85	39.67
Current→2090s	SSP1‐2.6	83.53	23.99	16.47
	SSP2‐4.5	73.45	28.59	26.56
	SSP3‐7.0	60.31	41.43	39.70
	SSP5‐8.5	46.97	38.84	53.03

Under the SSP1‐2.6 scenario, the total area of the potential suitable region for *Z*. *spinosa* changed slightly (98.86%–104.62% of the current corresponding value), and showed only a small contraction in the 2070s. The area of the high suitability region increased to varying degrees. Notably, the area of high suitability region in the 2090s was 2.89 × 10^4^ km^2^, with an increase of 201.26% compared with the current area.

Under the SSP2‐4.5 scenario, the total area of the suitable region for *Z*. *spinosa* showed a slight increase (102.03%–107.46% of the current corresponding value). The area of the highly suitable region initially increased, contracted, and then finally increased. The values during the three future periods were 1.55 × 10^4^ km^2^ (2050s), 0.94 × 10^4^ km^2^ (2070s), and 1.85 × 10^4^ km^2^ (2090s), accounting for 161.55%, 98.19%, and 192.78% of their current corresponding value, respectively.

Under the SSP3‐7.0 scenario, the total area of the suitable region for *Z*. *spinosa* increased to different degrees during the three future periods, and the total area of the suitable region (179.51 × 10^4^ km^2^) increased most significantly in the 2070s, which increased by 10.40% compared with the current value. The area with the high suitability region showed a trend of contraction‐expansion‐contraction, accounting for 161.55% (2050s), 98.19% (2070s), and 192.78% (2090s) of the current value, respectively.

Under the SSP5‐8.5 scenario, the total area of the suitable region of *Z*. *spinosa* initially increased and then contracted, and the area of the suitable region was only 139.53 × 10^4^ km^2^ in the 2090s. In the 2090s, the area of the moderate suitability region was 31.16 × 10^4^ km^2^ (only 38.23% of the current corresponding value). Except for the 2050s, the area of the high suitability region showed a gradual contraction trend. In the 2090s, the high suitability region was only 0.12 × 10^4^ km^2^ in area (12.82% of the current corresponding value).

### 3.5 Low impact area

**FIGURE 6:**
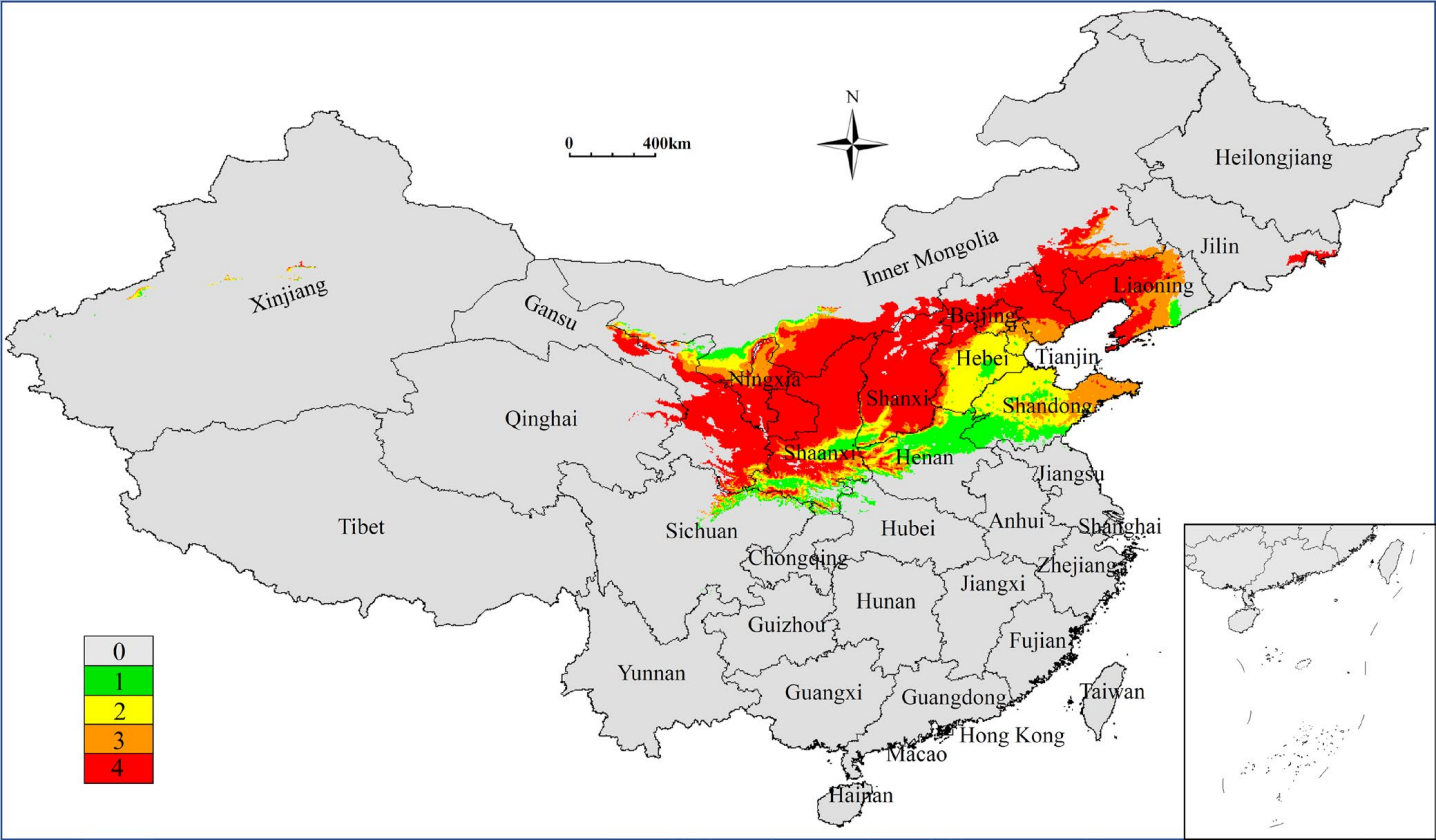
Composite prediction of low impact areas supported by varying numbers of shared socio‐economic pathways (SSP1‐2.6, SSP2‐4.5, SSP3‐7.0 & SSP5‐8.5)

The prediction of the low impact area differed under various climatic scenarios (Figure 6, Figure S1; Table 4). With increased climatic severity (SSP1‐2.6→SSP5‐8.5), the distribution of the low impact area for *Z*. *spinosa* decreased continuously (134.33 × 10^4^ km^2^→76.38 × 10^4^ km^2^). However, Shanxi, Ningxia, Central and Northern Shaanxi, Central and Eastern Gansu, Northern Hebei, Central and Western Liaoning, and Southern Inner Mongolia were always predicted to be low impact areas for the growth of *Z*. *spinosa* (Figure 6). Furthermore, the Jiaodong Peninsula of Shandong Province, Central and Western Liaoning Province, Northern Tianjin, Northern Ningxia Province, and Southern Shaanxi Province were classified as low impact areas under three climatic scenarios.

**TABLE 4:** Low impact areas (LIAs) under different shared socio‐economic pathways (SSPs)

LIA statistics	Shared socio‐economic pathways (SSPs)			
	SSP1‐2.6	SSP2‐4.5	SSP3‐7.0	SSP5‐8.5
Geographic area (×10^4^ km^2^)	134.33	119.42	98.06	76.38
Percentage of current suitable area (%)	82.61	73.44	60.30	46.97
Percentage of SSP1‐2.6 area (%)	100.00	88.90	73.00	56.86

### 3.6 Shift in the distribution center of the suitable region

The potential suitable region for *Z*. *spinosa* showed a trend of gradually shifting to the northwest under all climatic scenarios, except SSP1‐2.6. For example, under the SSP5‐8.5 scenario, the center of the potential suitable region shifted to the northwest by 183.64 km (2050s), 106.79 km (2070s), and 106.91 km (2090s) over time. The central point moved from Yushe County (Shanxi Province) currently, to the Ejin Horo Banner (Inner Mongolia) in the 2090s. Under the SSP1‐2.6 scenario, the center of the potential suitable region initially shifted to the northeast by 155.39 km (2050s), then to the northwest by 66.31 km (2070s), and finally to the northeast by 78.26 km (2090s) over time. The central point transitioned from Yushe County (Shanxi Province) to Wutai County (Shanxi Province).

## 4 DISCUSSION

### 4.1 Accuracy of model prediction

Using the MAXENT model to predict the distribution of potential suitable regions for species has become one of the commonly used research methods across the domains of biology, ecology, biogeography, biological evolution, and species conservation (Araújo & Guisan, 2006). However, previous studies often neglected the optimization of model parameters, or involved inadequate optimization, which could affect the accuracy of the predictive model (Li et al., 2020; Yan, Feng, Zhao, Feng, Wu, et al., 2020; Yan, Feng, Zhao, Feng, Zhu, et al., 2020). Previous studies revealed that the quality control of species occurrence points, screening of environment variables, selection of GCMs and SSPs, threshold selection, and the optimization of RM and FC all had significant effects on the predictive results of the model (Wei et al., 2021; Yang et al., 2021; Ye et al., 2020; Zeng et al., 2016). For this study, these parameters were systematically optimized so as to ensure predictive accuracy to the maximum extent.

### 4.2 Effects of environmental variables on species distribution

The geographical distribution of plants is restricted mainly by climatic variables, where hydrothermal conditions play a leading role in their distribution patterns (Sun et al., 2020). Precipitation is likely to increase or decrease as the climate changes and will affect soil moisture, which can cause plants to fail to reproduce, grow, and survive (Feng et al., 2020). Our study revealed that the main bioclimatic variables that affected the potential distribution of *Z*. *spinosa* were the mean temperature of the coldest quarter and the precipitation of the warmest quarter (Table 1). The mean temperature of coldest quarter for *Z*. *spinosa* is −9.1—5.8°C; it is much more likely to affect survival or cause a mismatch between phenology and season. The suitable range of the precipitation of the warmest quarter for *Z*. *spinosa* is 93–675 mm, which implies that *Z*. *spinosa* may prefer relatively dry environments. This was evidenced by our field surveys: *Z*. *spinosa* prefers arid and mild soil, and generally grows on sunny or semi‐sunny slopes, with little requirement for water or fertilizers. Furthermore, it is more suitable for temperate monsoon and temperate continental climates; thus, it appears that *Z*. *spinosa* is more suitable for planting in Northern China.

Under the SSP2‐4.5 and SSP3‐7.0 scenarios, the total area of the potential suitable regions for *Z*. *spinosa* increased by varying degrees. This indicated that within a certain range, the rising temperatures and increased rainfall caused by the greenhouse effect were more conducive for the growth of *Z*. *spinosa*. However, when the effects of global warming exceed the optimal tolerance range of species, the potential distribution region would also contract to varying degrees. For example, under the SSP5‐8.5 scenario, the potential suitable region of *Z*. *spinosa* decreased significantly in the 2070s and 2090s.

### 4.3 Changes in spatial patterns of potential suitable regions

**FIGURE 7:**
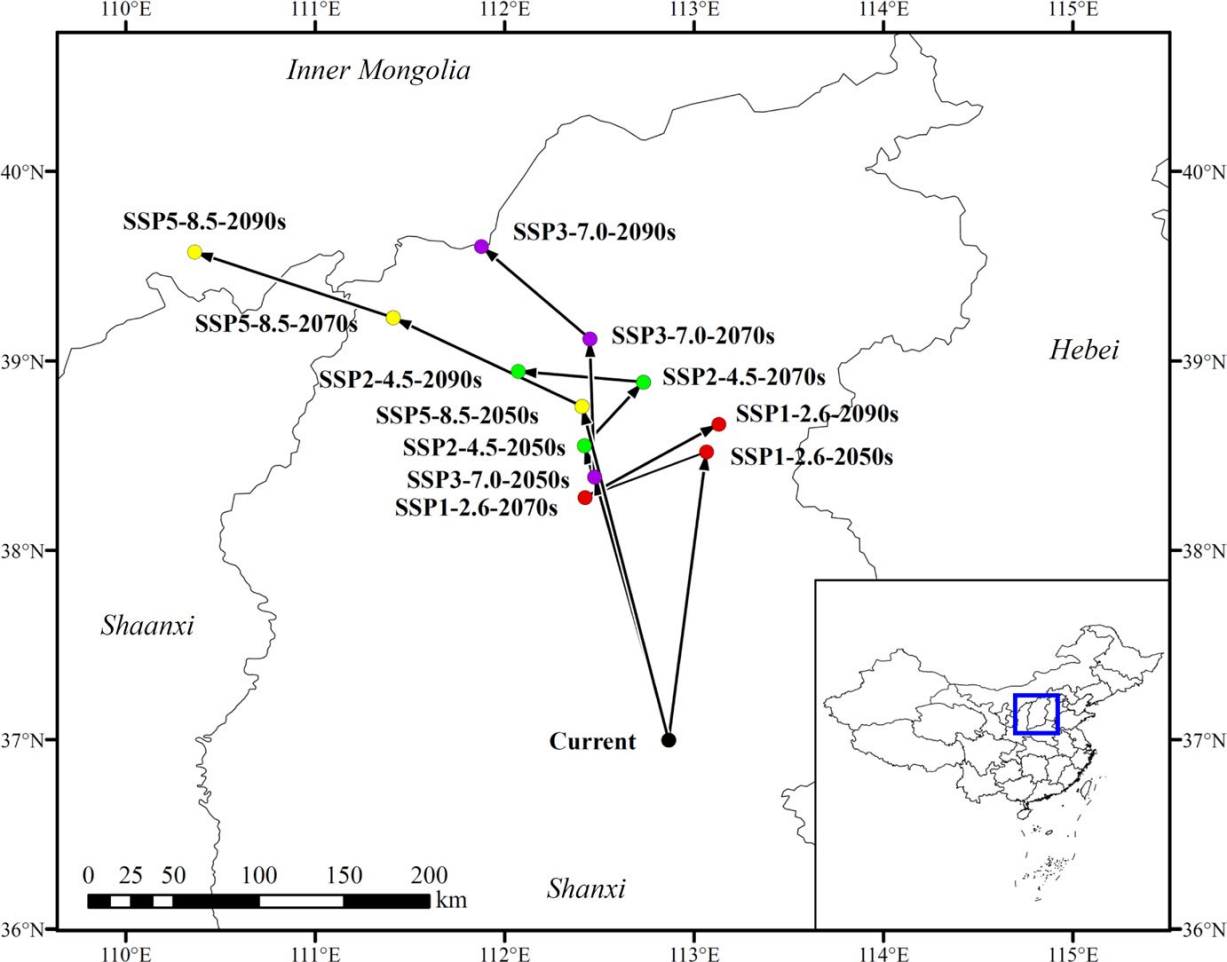
Core distribution shifts under 12 climate scenarios/years. Arrows indicate the magnitude and direction of predicted change over time

In general, over time, the potential suitable regions for *Z*. *spinosa* gradually migrated to high‐latitude areas (Figures 4 and 7). For example, under the SSP1‐2.6 scenario, the suitable region of *Z*. *spinosa* increased, except for the 2070s; however, the distribution area changed. Jiangsu, Guizhou, Hubei, Anhui, southern Henan, and adjacent regions were close to being no longer suitable for its growth. Meanwhile, there was an expansion of potentially suitable areas in Jilin, Liaoning, Inner Mongolia, Gansu, Qinghai, Xinjiang, and adjacent regions. Under the SSP5‐8.5 scenario, the suitable regions were further transferred to the north by the 2070s. Northeastern Qinghai, central and southern Shaanxi, southwestern Shanxi, central and southern Hebei, most parts of Henan, central and western Shanxi were predicted to no longer be suitable for *Z*. *spinosa*. By the 2090s, with the further intensification of the greenhouse effect, Shandong and Tianjin became unsuitable for the growth of *Z*. *spinosa*, whereas the area of potential suitable regions in Hebei, Beijing, and adjacent regions were also greatly reduced.

Compared with previous studies (Zhao et al., 2021), we also predicted the distribution of potential suitable areas in 2090s and the shift in the distribution center of the suitable region in different ages. We found that during the period of SSP5‐8.5‐2090s, the potential suitable area of *Z*. *spinosa* would shrink by 14.19%; this indicated that with the intensification of the greenhouse effect, when the average temperature line exceeded the tolerance limit of *Z*. *spinosa*, the distribution area of *Z*. *spinosa* would gradually decrease and move to higher latitude. Therefore, by using the prediction results of the model, it is possible to take reasonable protection measures to *Z*. *spinosa* as soon as possible, which would alleviate the impact of climate change. In addition, the total area of potential suitable areas of *Z*. *spinosa* tends to increase, and the expansion area is mainly located in middle and high latitudes, while the decrease area is mainly located in low latitudes, which is consistent with the view of Zhao, Zhang, et al. (2021).

### 4.4 Development and protection of germplasm resources

*Ziziphus spinosa* is a woody plant with high economic value that can tolerate cold and dry environments, and is suitable for planting in northern China (Li et al., 2021; Song et al., 2020). Furthermore, particularly in the Loess Plateau area, it can also be employed as a shelter forest species. Our study predicted that Shanxi, central and northern Shaanxi, eastern Gansu, central and southern Liaoning, northern Hebei, central and southern Ningxia, southern Inner Mongolia, and eastern Jilin would be less affected by climate change (Figure 6, Figure S1); thus, these areas would be suitable for increased *Z*. *spinosa* planting.

Due to anthropogenic destruction of habitat and climate change, wild *Z*. *spinosa* resources are gradually shrinking. According to the prediction results of the model, the potential suitable area of *Z*. *spinosa* will gradually shift to the north in the future. This means that future artificial planting area of *Z*. *spinosa* should be preferably established in the north of the Qinling Mountains (Figure 6), so as to alleviate the impact of climate change on the growth of *Z*. *spinosa*. In addition, with the aggravation of climate severity, the distribution of *Z*. *spinosa* in Shandong Province will gradually reduce, which may pose a challenge to the protection of local wild *Z*. *spinosa* resources. It may be necessary to establish a local germplasm bank of *Z*. *spinosa* in time.

Jujuboside A & B and Spinosin are the main medicinal ingredients of Ziziphi Spinosae Semen, and their content has become one of the standards through which to measure the quality of *Z*. *spinosa*. The current highly suitable regions for *Z*. *spinosa* growth are primarily restricted to Shanxi (Changzhi and Jincheng), Hebei (Xingtai, Handan and Baoding), Beijing, and adjacent regions (Figure 2). These regions are generally considered as the traditional and authentic production areas of *Z*. *spinosa* (Li et al., 2021). However, under the climate change, the quality of *Z*. *spinosa* may be negatively impacted in these areas. Therefore, the investigation, collection, and management of high‐quality germplasm and the establishment of core germplasm resource banks may play a key role in the protection of high‐quality *Z*. *spinosa* resources.

### 4.5 Study limitations

For this study, we considered only the effects of bioclimatic variables on species distribution, which was also practically influenced by a variety of biological factors (e.g., interspecific competition, predation, and disease) and abiotic factors (e.g., soil, topography, and anthropogenic activities) (Sun et al., 2020). Moreover, our study assumed that species would have a sufficient dispersal capacity to migrate to any climatically suitable area under climate change. However, we did not consider factors such as the species migration rate, as well as geographical and ecological isolation, all of which can lead to potential discrepancies between predicted and actual distributions. Elucidating the influences of all these factors requires a more comprehensive niche modeling approach, which has yet to be done in future studies (Wilting et al., 2010).

ENMs/SDMs make important assumptions about the relationship between species distributions and their environment that may limit their ability to accurately predict future species distributions. In particular, SDMs in theory assume stable fundamental niches, but in practice, they assume stable realized niches. The assumption of a fixed realized niche relative to climate variables remains unlikely for various reasons, particularly if novel future climates open up currently unavailable portions of species' fundamental niches (Veloz et al., 2012).

## 5 CONCLUSION

An optimized MAXENT model was employed to predict the distribution patterns and changes of potentially suitable *Z*. *spinosa* regions in China under different climate scenarios (SSP1‐2.6, SSP2‐4.5, SSP3‐7.0, and SSP5‐8.5) in recent (1970–2000) and future periods (2050s, 2070s, and 2090s). The potential future suitable area of *Z*. *spinosa* would expand except SSP‐1–2.6‐2070s, SSP‐5–8.5‐2070s, and SSP‐5–8.5‐2090s. Meanwhile, considering the distribution of *Z*. *spinosa* during different time periods and under variable climate change scenarios, the low impact areas were primarily distributed in Shanxi, Shaanxi, Ningxia, Gansu, Liaoning, Inner Mongolia, and Jilin Provinces. Furthermore, except for SSP1‐2.6, the center of the potential suitable region of *Z*. *spinosa* showed a trend of gradually shifting to the northwest. The findings of this study provide important references for the investigation and protection of germplasm resources and the promotion of artificial cultivation of *Z*. *spinosa*.

## CONFLICT OF INTEREST

The authors declare no competing interests.

## AUTHOR CONTRIBUTIONS

**Qian Zhao:** Conceptualization (lead); Data curation (lead); Software (lead); Writing – original draft (lead). **Ze‐Yuan Mi:** Formal analysis (equal); Investigation (equal); Visualization (supporting). **Chan Lu:** Data curation (equal); Investigation (equal). **Xin‐Fei Zhang:** Investigation (equal); Resources (supporting). **Li‐Jun Chen:** Data curation (equal); Formal analysis (equal). **Shi‐Qiang Wang:** Supervision (supporting); Writing – review & editing (equal). **Jun‐Feng Niu:** Conceptualization (equal); Funding acquisition (equal); Writing – review & editing (lead). **Zhe‐Zhi Wang:** Funding acquisition (equal); Methodology (equal); Validation (lead); Writing – review & editing (equal).

## Supporting information

Fig S1

## Data Availability

The data that support the findings of this study are openly available in Dryad at https://doi.org/10.5061/dryad.gqnk98snf.
