# Supplementary material for: Predicting potential distribution of Ziziphus spinosa (Bunge) H.H. Hu ex F.H. Chen in China under climate change scenarios
Source: Ecol Evol. 2022 Feb 17;12(2):e8629. doi: 10.1002/ece3.8629 (PMC8855015; doi:10.1002/ece3.8629)
Supplement: Supplementary file 1 — Fig S1 [file ECE3-12-e8629-s001.docx]

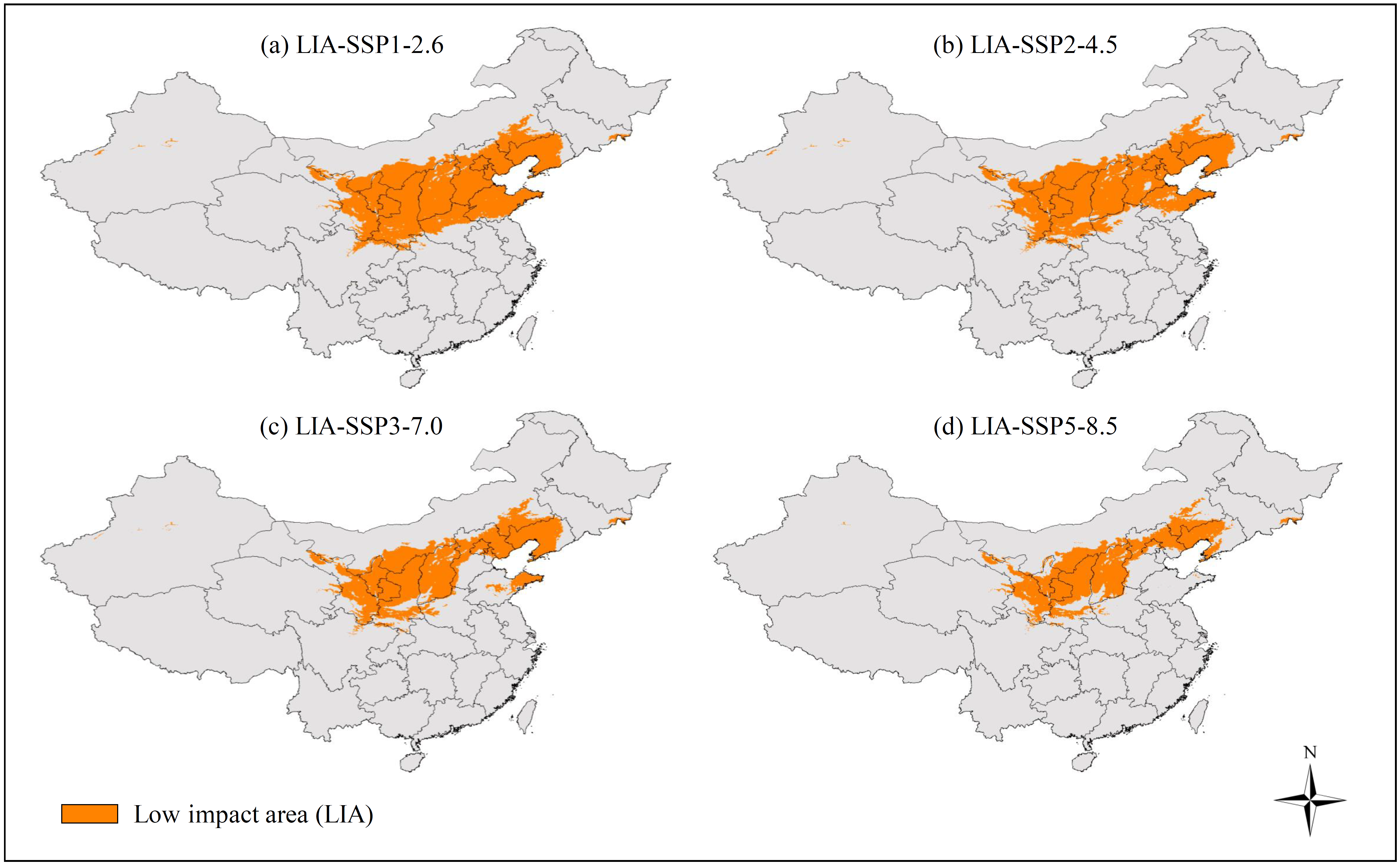


**Figure S1** Low impact area of *Ziziphus spinosa* under four shared socio-economic pathways (SSP1-2.6, SSP2-4.5, SSP3-7.0 & SSP5-8.5)
